# The Immune Defense Response and Immune-Related Genes Expression in *Macrobrachium nipponense* Infected with Decapod Iridescent Virus 1 (DIV1)

**DOI:** 10.3390/ani14192864

**Published:** 2024-10-04

**Authors:** Xiaojian Gao, Yujie Zhu, Qieqi Qian, Anting Chen, Lijie Qin, Xinzhe Tang, Qun Jiang, Xiaojun Zhang

**Affiliations:** College of Animal Science and Technology, Yangzhou University, Yangzhou 225009, China; gaoxj336@163.com (X.G.); zyj816020@163.com (Y.Z.); qieqi2000810@163.com (Q.Q.); chenanting2002@163.com (A.C.); 19352708430@163.com (L.Q.); txz001009@163.com (X.T.); jiangqun1013@163.com (Q.J.)

**Keywords:** *Macrobrachium nipponense*, DIV1, immune response, differently expressed genes, transcriptome analysis

## Abstract

**Simple Summary:**

Decapod iridescent virus 1 (DIV1), a new virus, has posed significant challenges to the *Macrobrachium nipponense* industry, and little is known about the mechanism of the host response to DIV1 infection. In order to understand the immune response of *M. nipponense* to DIV1 infection, transcriptome analysis was conducted on the hepatopancreas of *M. nipponense* to examine the global expression patterns at 48 hpi with DIV1. Our results showed that multiple immune-related genes (e.g., *lectin*, *dorsal*, *wnt6*, *hsp70*, *integrin* and *caspase*) may play a significant role in *M. nipponense* against DIV1 infection, and the immune-related signaling pathways were significantly activated. This study has the potential to enhance our comprehension of the immune response of *M. nipponense* to DIV1 infection, which is advantageous for future treatment strategies for disease caused by DIV1.

**Abstract:**

*Macrobrachium nipponense* is a significant cultivated species in China. However, decapod iridescent virus 1 (DIV1), as a newly discovered crustacean-lethal virus, has resulted in significant financial losses for the *M. nipponense* industry. In order to examine the immunological response of *M. nipponense* to DIV1, we conducted transcriptome analysis of the hepatopancreas from *M. nipponense* infected with DIV1 using RNA-seq. RNA sequencing analysis identified a combined total of 41,712 assembled unigenes, and 7014 genes that showed differential expression were identified in the group infected with DIV1, compared to the control group. Among these DEGs, 3952 were found to be up-regulated, while 3062 were down-regulated; many well-characterized DEGs were involved in innate immune defense, particularly involving the C-type lectin receptor signaling pathway, complement and coagulation cascades, phagosome, lysosome and PPAR signaling pathway. Moreover, the expression levels of well-known immune-related genes (*dorsal*, *wnt6*, *lectin*, *caspase*, *integrin*, *hsp70*) in the hepatopancreas and hemolymph were investigated by Quantitative real-time PCR (qRT-PCR), and the findings demonstrated a significant increase in gene expression in the hepatopancreas and hemolymph at various time points after infection. The results acquired in this study offered further comprehensive understanding of the immunological response of *M. nipponense* to DIV1 infection.

## 1. Introduction

*Macrobrachium nipponense* (the oriental river prawn) is widely distributed in freshwater and estuarine areas of China and several other Asian nations; it is one of the most economically important farmed prawns in China [1], and the annual output reached 226,392 tons in 2023 [2]. Due to the gradual expansion of culturing, inadequate management practices, deterioration of water quality and environmental stress, *M. nipponense* faced several threats from bacterial pathogens in the last few years, such as *Aeromonas hydrophila* [3], *Aeromonas veronii* [4], *Aeromonas salmonicida* [5], *Vibrio cholerae* [6], *Vibrio mimicus* [7] and *Citrobacter freundii* [8]. Recently, there has been an epidemic of novel viral disease caused by decapod iridescent virus 1 (DIV1) in *M. nipponense*, which has induced a significant number of deaths and substantial financial losses for the *M. nipponense* farming industry [9,10].

DIV1 is a recently identified virus that has an enveloped icosahedral structure, which was found earlier in *Cherax quadricarinatus* (named *C. quadricarinatus* iridovirus, CQIV CN01) [11] and *Penaeus vannamei* (named shrimp hemocyte iridescent virus, SHIV 20141215) [12]. To date, previous studies showed that DIV1 has been identified as a new infectious virus found in many commercially farmed crustaceans, including *Macrobrachium rosenbergii* [13], *Marsupenaeus japonicus* [14], *Procambarus clarkia* [15], *P. vannamei* [16], *Penaeus monodon* [17], *Exopalaemon carinicauda* [18] and *Portunus trituberculatus* [19], and has caused huge losses of crustacean aquaculture. Our previous study also revealed that *M. nipponense* is a susceptible host to DIV1 infection, and DIV1 mainly infects the hepatopancreas and hemocytes of *M. nipponense* [9]. To date, much research on DIV1 has focused on etiology, pathology, epidemiology and detection, but little is known about the molecular mechanism of the host in response to DIV1. Hence, it is essential to acquire a more comprehensive comprehension of the host–pathogen interaction in order to devise efficacious preventative and management strategies for the disease induced by DIV1.

Similar to other crustaceans, *M. nipponense* mainly relies on its innate immune system to prevent pathogens’ invasion [20]. The hepatopancreas of crustaceans is a crucial organ integrated into the immune and metabolic functions and usually is the primary target organ attacked by pathogenic microorganisms [21]. Transcriptome sequencing is a highly effective technology for identifying immune-related genes and exploring the immune mechanisms of crustaceans in relation to pathogenic microorganisms [22], and the hepatopancreas has been widely used in transcriptome analysis of crustaceans in response to virus infections [23]. However, to date, there has been no transcriptomic study on the response of *M. nipponense* to DIV1 infection. In the present study, we used high-throughput RNA sequencing to develop *M. nipponense* hepatopancreas transcriptomes after infection by DIV1. Subsequently, qRT-PCR was used to determine the expression patterns of selected immune-related genes in *M. nipponense* at various time intervals following DIV1 infection. This study has the potential to enhance our comprehension of the immune response of *M. nipponense* to DIV1 infection, which is advantageous for future treatment strategies of disease caused by DIV1.

## 2. Materials and Methods

### 2.1. Prawns Culture and DIV1 Infection Experiment

A total of 500 experimental *M. nipponense* (2.71 ± 0.13 g) were acquired from a farm located in Jintan county, Jiangsu province, China. Prior to the DIV1 challenge experiment, the prawns were acclimated for a week in aerated and filtered 28 °C water, and 10 of the prawns were randomly selected for confirmation of freedom from pathogens, including infectious hypodermal and hematopoietic necrosis virus (IHHNV), white spot syndrome virus (WSSV) and DIV1 by PCR or RT-PCR; the specific primers employed for virus detection were according to the previous study of Qian et al. [13].

DIV1 inoculum was prepared from *M. nipponense* artificially pre-infected with the isolate DIV1-mn [9]; shell-off cephalothorax of DIV1-infected *M. nipponense* was homogenized in PBS and centrifuged at 8000 rpm (4 °C, 20 min) to obtain the supernatant, filtered by 0.22-μm filter, and the amount of viral load was measured as 2.14 × 10^8^ copies/mL by real-time PCR performed according to the methods of Qiu et al. [24]. Subsequently, the healthy *M. nipponense* were randomly divided into DIV1-infected and control groups. The prawns in DIV1-infected were injected intramuscularly with 50 µL of DIV1 inoculum (2.14 × 10^4^ copies/mL), the control group was given an injection of 50 µL of PBS (0.01 M, pH 7.4), and each group contained three repetition tanks (50 prawns per tank). After 48 h post-injection (hpi), the hepatopancreas from three individuals in each tank were combined as one sample, each group had three parallel samples, and the samples were rapidly frozen in liquid nitrogen for RNA extraction.

### 2.2. cDNA Library Construction, and Transcriptome Sequencing

Total RNA was isolated individually from the hepatopancreas of both the DIV1-infected and control groups using TRIzol^®^ Reagent (Invitrogen, Carlsbad, CA, USA). The RNA quality was assessed using a 2100 Bioanalyzer (Agilent, Santa Clara, CA, USA), and its quantity was determined using NanoDrop 2000. The mRNA-seq library was constructed using a TruSeqTM RNA sample preparation Kit from Illumina (San Diego, CA, USA). Briefly, the mRNA was extracted from total RNA using polyA selection with oligo(dT) beads and then fragmented into randomly short pieces using a fragmentation buffer. The cDNA synthesis, end repair, poly(A) addition and ligation of the Illumina-indexed adaptors were carried out following the Illumina procedure. Following the selection of cDNA target fragments ranging from 200 to 300 bp, PCR amplification was performed, and then the cDNA libraries were sequenced using the Illumina NovaSeq 6000 platform (150 bp × 2, Shanghai Biozeron Biotechnology Co., Ltd., Shanghai, China).

### 2.3. Transcriptome Assembly and Functional Annotation

The high-quality clean reads of DIV1-infected and control groups were acquired by removing the raw reads with adapters, ambiguous “N” nucleotides, low quality reads and rRNA using Trimmomatic software (v0.39) [25]. Then, the clean data from all samples were used for RNA assembly using Trinity with default parameters [26]. TGICL software (v2.1) was used to remove redundant sequences and generate unigenes [27]. The assembled transcripts were annotated in the following databases for functional analysis, including NCBI protein nonredundant (NR) [28], Swiss-Prot [29], Clusters of Orthologous Groups of proteins (KOG) [30], Kyoto Encyclopedia of Genes and Genomes (KEGG) [31] and Gene Ontology (GO) [32].

### 2.4. Screening and Enrichment Analysis of Differentially Expressed Genes (DEGs)

To identify significantly differential expressed genes (DEGs) between DIV1-infected and control groups, the gene expression levels were calculated by the reads per kilobase of exon per million mapped reads (RPKM) method. RSEM was used to quantify gene and isoform abundances [33], and EdgeR was used for screening DEGs [34]. The significant DEGs were defined based on log2 (FoldChange)| ≥ 1 and a cutoff false discovery rate (FDR) ≤ 0.05. The Gene Ontology (GO) enrichment analysis of DEGs was performed by Blast2GO, and Kyoto Encyclopedia of Genes and Genomes (KEGG) enrichment analysis of DEGs was performed by KOBAS.

### 2.5. RNA-Seq Data Validation

To validate the reliability of the RNA-seq data, eight randomly selected DEGs were conducted for qRT-PCR analysis using the identical RNA samples as for the RNA-Seq, and the primer sequences are listed in Table 1. Briefly, 1 μg of RNA was used to generate cDNA using TransScript One-Step gDNA Removal and cDNA Synthesis Supermix (Vazyme Biotech Co., Ltd., Nanjing, China). The qRT-PCR was conducted by using a Thermofisher QuantStudio Real-Time PCR System. The qRT-PCR reactions consisted of 10 μL of 2 × ChamQ Universal SYBR qPCR Master Mix (Vazyme), 1 μL of cDNA, 0.4 μL of forward and reverse primers (10 mM), 9.2 μL of ddH_2_O. The amplification procedures were performed according to the following conditions: 95 °C for 30 s, 40 cycles of 95 °C for 10 s and 60 °C for 30 s. The average Ct values were normalized by 18S rRNA as the reference gene, and the relative expression level of the target gene were calculated according to the 2^−ΔΔCt^ method described by Livak and Schmittgen; all reactions were performed in triplicates [35].

### 2.6. Detection of Immune-Related Gene Expression after Different Hours Post-Infection

According to our RNA-seq results, the six up-regulated immune-related genes (dorsal, wnt6, lectin, caspase, integrin, hsp70) were selected for detection of the expression levels in the hepatopancreas and hemocytes of *M. nipponense* at different times post-infection with DIV1 by using qRT-PCR to examine the immune response. In the immune challenge experiments, the prawns were injected intramuscularly with 50 μL of DIV1 inoculum (2.14 × 10^4^ copies/mL) as the challenge group, and the prawns in control group received an identical amount of sterilized PBS by intramuscular injection. Subsequently, samples of the hepatopancreas and hemocytes were collected at 12, 24, 48, 72 and 96 h post-infection (hpi) in order to extract RNA. The qRT-PCR reactions and amplification protocols and relative expression calculation are described in Section 2.5.

### 2.7. Statistical Analysis

The significant differences among the DIV1 infected and control groups were analyzed by *t*-test using GraphPad Prism 10 software. The data were expressed as means ± SD; * *p* < 0.05 represents significant; ** *p* < 0.01 represents highly significant.

## 3. Results

### 3.1. Transcriptome Sequencing and De Novo Assembly

Comparing the transcriptomic profile of hepatopancreas samples collected from DIV1-infected and control groups was performed by RNA-seq, which can provide a comprehensive insight into revealing the immune response during early infection of DIV1 in *M. nipponense*. After eliminating out low-quality reads, transcriptome sequencing generated 38,774,382–41,367,100 clean reads from the six samples (Table 2). The transcriptome data in this study are available in NCBI Sequence Read Archive (SRA) repository under BioProject accession number PRJNA1086475.

### 3.2. Functional Annotation and Classification of M. nipponense Transcriptome Sequences

**Figure 1:**
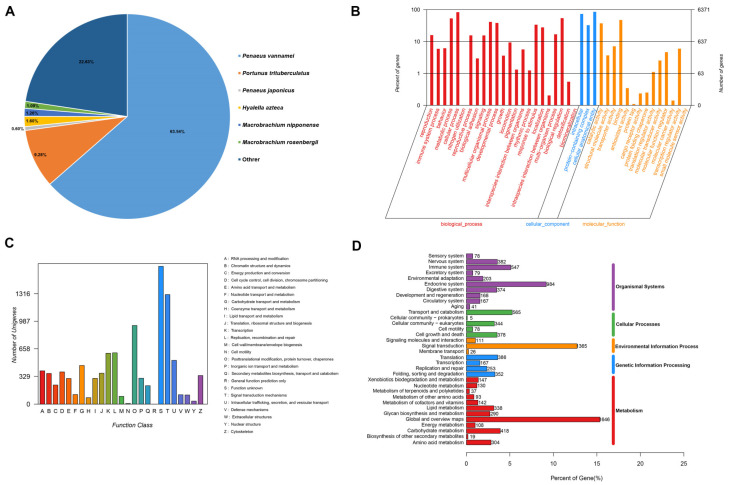
Sequence analysis and functional annotation of unigenes from *M. nipponense* under DIV1 infection. (**A**) Map of species distribution in the NR database; (**B**) GO classifications of assembled unigenes; (**C**) KOG function classification of unigenes; (**D**) KEGG annotation of unigenes.

In order to comprehensively annotate the functional information of the assembled sequences, a total of 41,712 unigenes were found to have significant matches when compared to the known sequences in the databases, including the NR (30.61%), GO (15.27%), KOG (22.33%), KEGG (42.26%), and SwissProt (17.63%) databases (Table 3). NR had the largest number of homologous sequences in the assembled transcripts, with 12,770 transcripts annotated in the database; the top five species distribution with the highest similarity in the NR database were *P. vannamei* (63.54%), *P. trituberculatus* (9.28%), *Hyalella Azteca* (1.60%), *M. nipponense* (1.26%), *M. rosenbergii* (1.09%) (Figure 1A). Analysis using GO annotation indicated that 6371 unigenes were aligned into three categories (Figure 1B). In the biological process of GO annotation, most unigenes were enriched in “cellular process” (5299 unigenes) and “biological regulation” (3433 unigenes); in the cellular component, “cellular anatomical entity” (5436 unigenes) and “intracellular” (4664 unigenes) were the dominant subcategories; in the molecular function category, “binding” (3016 unigenes) and “catalytic activity” (2370 unigenes) were the most represented subcategories. The unigenes were also annotated using the KOG database, and a total of 9313 unigenes were annotated with 25 specific categories (Figure 1C). The most abundant categories were “Function unknown” (5303 unigenes) and “Signal transduction mechanisms” (1305 unigenes). To identify biochemical metabolism and signal pathways of the annotated unigenes, 5416 unigenes were annotated using the KEGG database (Figure 1D); the largest subcategory groups were “Global and overview maps” (1646 unigenes), “Signal transduction” (1365 unigenes), “Endocrine system” (984 unigenes), “Transport and catabolism” (565 unigenes) and “Immune system” (547 unigenes).

### 3.3. Identification of DEGs Related to DIV1 Infection

**Figure 2:**
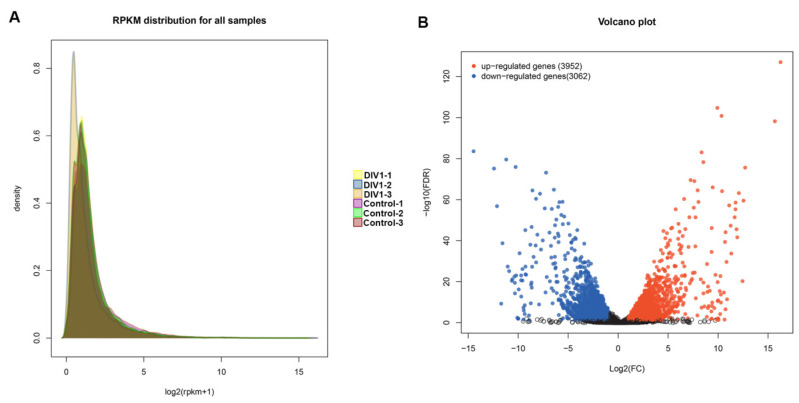
(**A**) RPKM density distribution. (**B**) Volcano depicting DEGs identified in the transcriptomes of *M. nipponense* hepatopancreas between DIV1-infected and control groups. Red circles represent up-regulated genes, blue circles represent down-regulated genes, and black circles indicate no DEGs.

To analyze and characterize the DEGs in *M. nipponense* associated with responding to DIV1 infection, transcriptome data from DIV1-infected and control groups were analyzed and compared. Gene expression levels in each sample were determined by the value of transcripts RPKM, and the overall distribution of gene expression is shown in Figure 2A. There were a total of 7014 DEGs screened in the DIV1-infected groups compared to the control group, including 3952 up-regulated genes and 3062 down-regulated genes, with log2(FoldChange)| ≥ 1 and FDR ≤ 0.05 as criteria for differences (Figure 2B). These DEGs were classified into pattern recognition receptors (PRRs), antimicrobial peptides (AMPs) and other immunity-related proteins. Among these DEGs, some well-characterized genes involved in innate immune defense, e.g., dorsal, wnt6, lectin, caspase, integrin, heat shock protein (hsp70), were up-regulated significantly in the DIV1-infected groups (Table 3). Overall, undergoing DIV1 infection had a major effect on the entire gene expression profile of *M. nipponense*.

### 3.4. GO and KEGG Enrichment Analysis of DEGs in Response to DIV1 Infection

**Figure 3:**
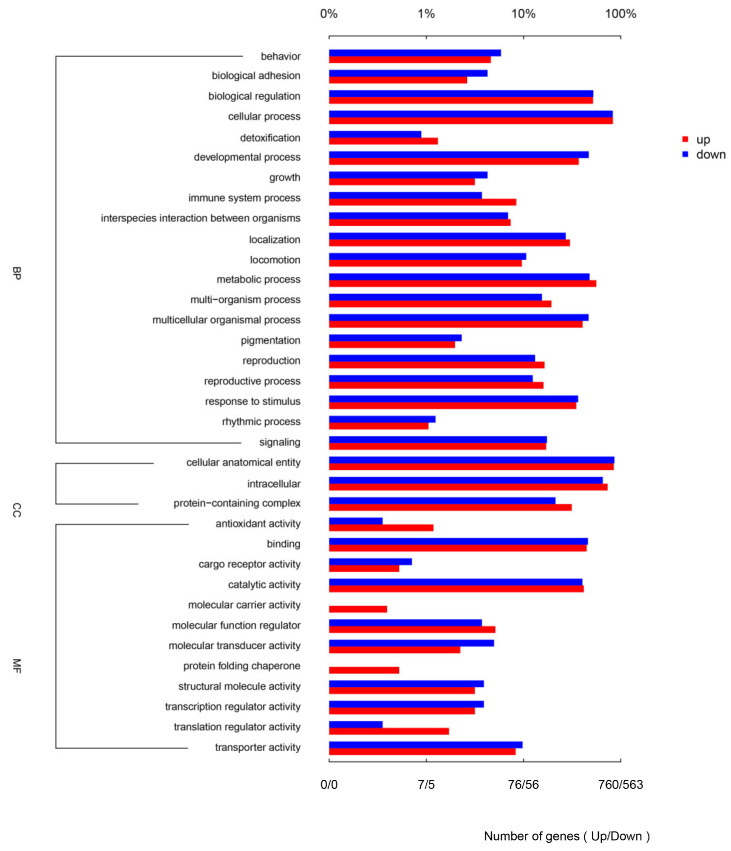
GO analysis of differential expression genes of the DIV1-infected and control groups.

In order to further investigate the DEGs in the immune response of *M. nipponense* against DIV1 infection, all the DEGs were annotated with the term in the GO and KEGG databases. In the GO enrichment analysis, a total of 240 DEGs were associated with three major functional classes, including biological processes (19 terms), molecular functions (12 terms) and cellular components (three terms). The largest GO subcategories of DEGs were “cellular processes”, “cellular anatomical entity”, “metabolic process”, “intracellular” and “binding”, and the abundant DEGs were also enriched in immune-related GO subcategories, such as “response to stimulus”, “immune system process” (Figure 3).

**Figure 4:**
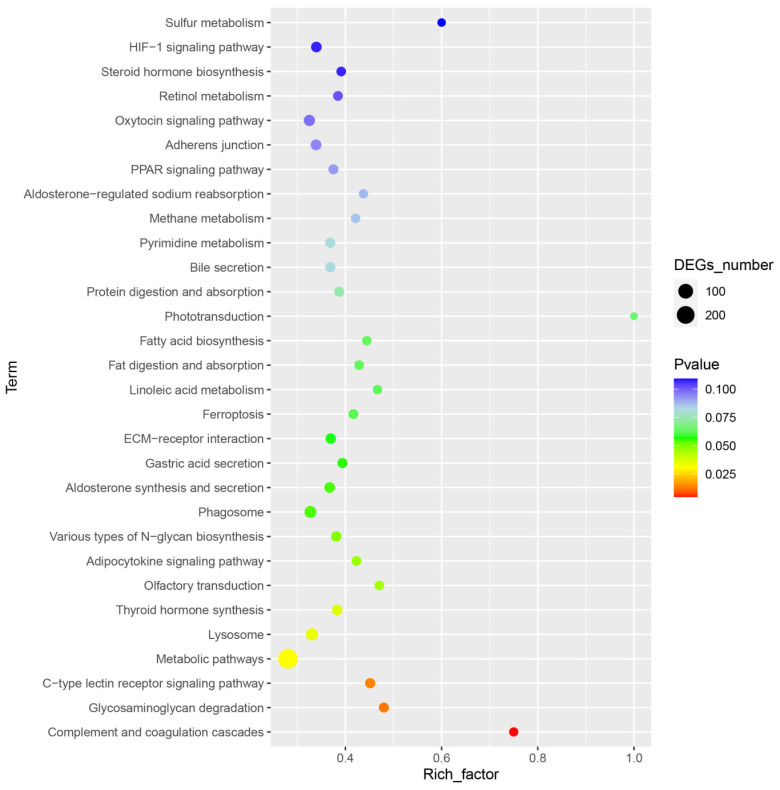
The top 30 KEGG enrichment pathways for DEGs; the rich factor refers to the ratio of the number of DEGs in the pathway and the number of all annotated genes in the pathway.

For further analysis of these DEGs, the DEGs also had been analyzed by KEGG enrichment, and a total of 253 KEGG pathways were significantly enriched. The top 30 enriched pathways associated with DIV1 infection at 48 hpi are presented in Figure 4; several significantly enriched pathways were related to immune response, including “C-type lectin receptor signaling pathway”, “Complement and coagulation cascades”, “Phagosome”, “Lysosome” and PPAR signaling pathway. In addition, many other well-known pathways related to immune response were shown in this study, such as apoptosis, the AMPK signaling pathway, Toll-like receptor signaling pathway, Wnt signaling pathway, Toll and Imd signaling pathway, etc.

### 3.5. Verification of the DEGs by qRT-PCR

**Figure 5:**
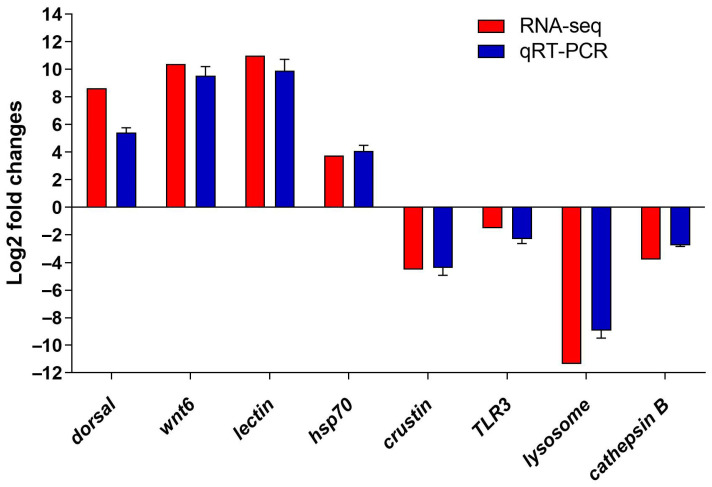
Validation of DEGs by qRT-PCR.

To validate the accuracy of the DEGs data from the RNA-seq results, four up-regulated DEGs (*dorsal*, *wnt6*, *lectin*, *hsp70*) and four down-regulated DEGs (crustin, TLR3, lysosome, cathepsin B) were selected for qRT-PCR validation. The trend of qRT-PCR results was consistent with the sequencing results, which confirmed that the expression of the DEGs from transcriptome results were reliable (Figure 5).

### 3.6. Expression Profiles of Immune-Related Genes Expression after Different Hours Post-Infection

#### 3.6.1. Immune-Related Gene Expression in Hepatopancreas after Different Hours Post-Infection

**Figure 6:**
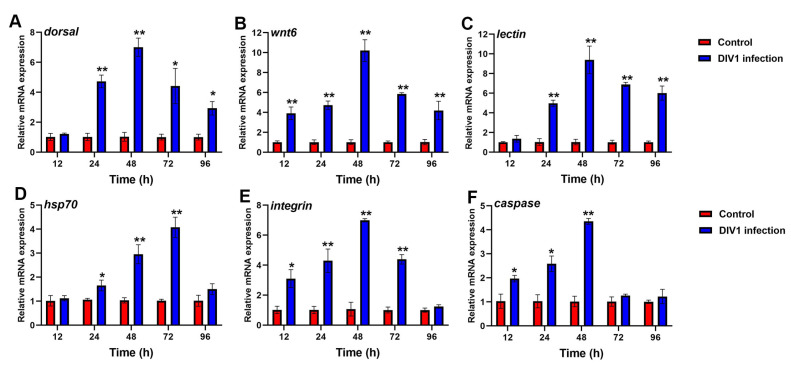
Analysis of immune-related genes expression in *M. nipponense* hepatopancreas after DIV1 infection. (**A**) *dorsal*, (**B**) *wnt6*, (**C**) *lectin*, (**D**) *hsp70*, (**E**) *integrin*, (**F**) *caspase*. Data are shown as the mean ± SD (*n* = 3) with error bars representing the standard errors, and significant differences are indicated by asterisk, * *p* < 0.05, ** *p*< 0.01, compared with the control group.

To further investigate the immune response in *M. nipponense* against DIV1 infection, the expression profiles of immune-related genes in the hepatopancreas of infected and control groups were detected by qRT-PCR. As shown in Figure 6, significant changes of *dorsal*, *wnt6, lectin*, *caspase*, *integrin* and *hsp70* were detected in the hepatopancreas of DIV1-infected groups compared to control groups. An increased expression of *dorsal* and *lectin* were observed in DIV1-infected prawns from 24 and 96 hpi, and reached a peak of 7.01, 9.39-fold at 48 hpi, respectively (Figure 6A,C). The expression of *wnt6* and *integrin* was significantly up-regulated from 12 to 72 hpi and reached a maximum of 10.20 and 6.99-fold at 48 hpi (Figure 6B,E). The expression of *hsp70* was significantly up-regulated from 24 to 72 hpi, reached peak of 4.08-fold at 72 hpi, and then decreased at 96 hpi (Figure 6D). The expression of *caspase* showed an up-regulation from 12 to 48 hpi, and the highest expression level was 4.36-fold at 48 hpi (Figure 6F).

#### 3.6.2. Immune-Related Gene Expression in Hemocytes after Different Hours Post-Infection

**Figure 7:**
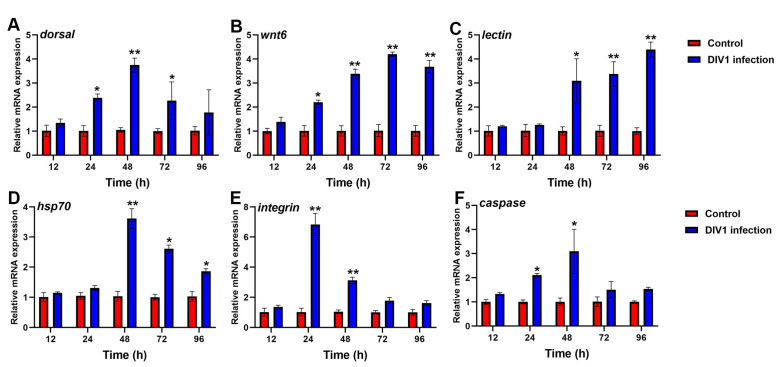
Analysis of immune-related genes expression in *M. nipponense* hemocytes after DIV1 infection. (**A**) *dorsal*, (**B**) *wnt6*, (**C**) *lectin*, (**D**) *hsp70*, (**E**) *integrin*, (**F**) *caspase*. Data are shown as the mean ± SD (*n* = 3) with error bars representing the standard errors, and significant differences are indicated by asterisk, * *p* < 0.05, ** *p*< 0.01, compared with the control group.

The transcript levels of *dorsal*, *wnt6*, *lectin*, *caspase*, *integrin* and *hsp70* in hemocytes showed significantly increased expression in DIV1-infected groups (Figure 7). Of these genes, higher expression of *dorsal* (3.75-fold), *hsp70* (3.62-fold) and *caspase* (3.09-fold) were observed in hemocytes of the DIV1 infection at 48 hpi (Figure 7A,D,F). The expression of *wnt6* was significantly increased from 24 to 96 hpi and reached a peak of 4.20-fold at 72 hpi (Figure 7B). *Lectin* was significantly up-regulated from 48 to 96 hpi and reached a maximum of 4.39-fold at 96 hpi (Figure 7C). The expression of integrin was found to be highest up-regulated (6.83-fold) at 24 hpi (Figure 6E).

## 4. Discussion

*M. nipponense* is an important economic indigenous aquaculture species and widely distributed in China [36]. However, the prawn-farming industry has suffered a great threat from DIV1 infections in recent years, which have caused significant economic losses to the *M. nipponense* industry [9,10]. Our previous study showed DIV1 could affect the hepatopancreas tissue and hemocytes of shrimp [9], but the research on the effects of DIV1 on the physiological function of the *M. nipponense* hepatopancreas is little known. In this study, transcriptome analysis was conducted on the hepatopancreas of *M. nipponense* to examine the global expression patterns at 48 hpi with DIV1 to reveal the immune response of *M. nipponense* to DIV1 infection. Our results showed that a multitude of essential immune genes and critical pathways were derived from the transcriptome data, including induced expressions of pattern recognition receptors (PRRs), activated signal transduction pathways, regulated immune systems and apoptosis, etc., which provided insight into the defense responses against DIV1 infection in *M. nipponense*.

*M. nipponense*, as with other crustaceans, lacks adaptive immunity and can only defend by non-specific immunity [20]. The hepatopancreas is a significant organ that participates in the immune defense function of crustaceans [21,23]. In this study, transcriptome analysis on the hepatopancreas of *M. nipponense* at 48 hpi found that a total of 7014 DEGs were identified, containing 3952 up-regulated genes and 3062 down-regulated genes. In order to obtain a deeper comprehension of the immunological response in the hepatopancreas of *M. nipponense* during DIV1 infection, the DEGs were mapped in the KEGG databases. The KEGG pathway analysis of DEGs also indicates significant changes in immune-related pathways such as the C-type lectin receptor signaling pathway, complement and coagulation cascades, phagosome and lysosome. Clearly, these KEGG pathways are all classical and widely reported in the immune response of crustaceans against pathogen infections [37]. Recently, several transcriptome analyses of other crustaceans infected with DIV1 were reported; transcriptome analysis of the hemocyte in *F. merguiensis* at 48 hpi with DIV1 showed that a total of 1003 DEGs were screened, including 929 up-regulated genes and 74 down-regulated genes, and many DEGs were enriched in lysosome, phagosome and the MAPK signaling pathway, Wnt signaling pathway and Toll-like receptor signaling pathway, which played important roles in its resistance to infections, which was similar to our study [38]. In a transcriptomics analysis of the hemocyte in *P. monodon* at 24 hpi with DIV1, the results showed that 6236 DEGs were identified, including 3140 up-regulated and 3096 down-regulated genes, and many DEGs were also enriched in the Toll and Imd signaling pathway, PI3K-Akt signaling pathway, NF-kappa B signaling pathway, NOD-like receptor signaling pathway, Lysosome and MAPK signaling pathway [17]. In the transcriptome research of hemocyte in *M. japonicus* under DIV1 infection at 24 h, half of the top 20 KEGG pathway enrichments were found to be immune-related pathways, including the Toll and Imd signaling pathway, IL-17 signaling pathway, C-type lectin receptor signaling pathway [14]. It was worth noting that almost all of these immune-related pathways were promoted after DIV1 challenge, which reflects the fierce immune response of the hepatopancreas during DIV1 infection. The results showed that the immune-related signaling pathways in the hepatopancreas were significantly activated under DIV1 challenge.

The immune-related genes of crustaceans also have an important role in immunity and the evolution of the immune response to pathogens infection [39]. In this study, it was shown that many potential immune-related genes were significantly up-regulated, such as lectin, dorsal, wnt6, hsp70, integrin and caspase. Hence, changes in the expression of these immune-related genes in the hepatopancreas and hemocytes of *M. nipponense* at different times post-infection with DIV1 were determined by using qRT-PCR. The first stage of the innate immune response involves the identification of pathogenic microorganisms via the use of PRRs [40]. As important PRRs, lectins have significant functions in the innate immune system of crustaceans by participating in various immune responses [41]. Previous studies showed that the transcription of C-type lectins were considerably heightened in the hepatopancreas of *M. japonicus* in response to WSSV infection and could directly interact with several WSSV envelope proteins [42]. Similarly, the expression of the *lectin* gene was significantly up-regulated after DIV1 infection in our study. In addition, pathogen infections can induce diverse host humoral and cellular activities via many signaling transduction pathways. Dorsal is a member of the NF-κB family found in crustaceans, which has a role in controlling the production of several immune effector proteins that possess antibacterial and antiviral properties [43]. Huang et al. [44] report that the expression of *MrDorsal* was markedly increased 48 h following WSSV infection, which was similar to our study. The Wnt family of genes has been implicated in the innate immune responses of aquatic animals [45]; Wang et al. [46] reported that the two Wnt homologues (*Mn-Wnt4* and *Mn-Wnt16*) transcripts evidently increased after bacterial and viral infection, and the expression of the *wnt6* gene was significantly enhanced in our study. Previous studies have shown that aquatic animals can up-regulate a series of heat shock proteins (HSPs) genes against pathogenic microorganism infections [47]. The expression of Hsp70 was increased as a part of an immune response against WSSV, IHHNV, *Vibrio harveyi* infection in shrimps [48], which was consistent with this study. In crustaceans, integrins have been found to have an important role in the innate immune system [49]; previous studies showed that β-integrin was up-regulated and activated the integrin-related signaling pathways to assist with WSSV infection in shrimps [50]. Our data also showed that integrin mRNA expression was significantly up-regulated after DIV1 infection. In invertebrates, caspases are central effectors in apoptosis which are involved in many metabolic processes, including development, metamorphosis and immune responses. Caspase was significantly up-regulated in this study; similarly, IHHNV infection leads to up-regulation of LvCAP-3 expression in *P. vannamei* [51]. The results showed that lectin, dorsal, wnt6, hsp70, integrin and caspase have a significant impact in *M. nipponense* against DIV1 infection.

## 5. Conclusions

In conclusion, this study for the first time performed a *M. nipponense* hepatopancreas transcriptomics analysis for DIV1 infection. The results showed that a total of 7014 DEGs were expressed in the DIV1-infected group, the immune-related signaling pathways were significantly activated, and *lectin*, *dorsal*, *wnt6*, *hsp70*, *integrin* and *caspase* may have a significant role against DIV1 infection. The results will enhance our comprehension of the molecular processes of the immunological response to DIV1 infection in *M. nipponense*.

## Figures and Tables

**Table 1:** qRT-PCR primers used in this study.

Target Gene	PCR Primers Sequence (5′-3′)	Product Size (bp)	Transcript ID
18S rRNA	CAGGCTTATGTTGTCTTGAA	159	TRINITY_DN6546_c0_g1
	CTATGTTGGATGTTGCTGTT		
*dorsal*	TATCTTCTTCTTCGGCAGTT	240	TRINITY_DN6071_c0_g1
	TACGGCTCCTCCATATTCT		
*wnt6*	TCCTAGAAGAGAAGTTCCTTAG	197	TRINITY_DN40081_c0_g1
	ACGCAGTCCTATGTTCCT		
*lectin*	AACGGTTGCTTCAGAGAA	203	TRINITY_DN7682_c0_g2
	CTTCGCCAGGAGTTATCTT		
*caspase*	AGACTCTGCGAACAACTC	199	TRINITY_DN1075_c0_g2
	TCTCCTCTTGTGCTCCAT		
*integrin*	TGGAGGCAATATCATCTGTT	245	TRINITY_DN7473_c0_g1
	CGTTACTCTTGGTGTTATAGG		
*hsp70*	TGCTTCACACGACTCTTC	213	TRINITY_DN3715_c0_g1
	CACCATCACCAACGACAA		
*crustin*	AGATGCGATGTTGCGTAT	107	TRINITY_DN12166_c0_g1
	AACCTCTGTGCTATGCTATA		
*TLR3*	TTGGTTATCGGACACATTCA	145	TRINITY_DN39664_c0_g1
	TGGCATCACTGGCATAGA		
*lysosome*	AATGCGATAGTTGCGATTG	194	TRINITY_DN5716_c0_g2
	GATGCTTCCAGTTCTTGTAG		
*cathepsin B*	ATACTGGACCGTGGCTATA	102	TRINITY_DN608_c0_g1
	CCTGGAGAATACTGGACATT		

**Table 2:** Statistics of shrimp *M. nipponense* hepatopancreas transcriptome sequences.

Groups	Raw Reads	Clean Reads	Q20 (%)	Q30 (%)	GC (%)
DIV1-1	41,164,510	40,917,484	98.93	96.05	44.15%
DIV1-2	39,738,020	38,774,382	98.11	95.96	45.32%
DIV1-3	40,491,956	40,883,392	98.96	96.16	45.23%
Control-1	41,630,056	41,367,100	98.44	96.56	44.71%
Control-2	39,738,020	39,593,902	98.73	95.21	44.68%
Control-3	40,491,956	40,285,660	98.80	96.61	45.55%

**Table 3:** Differentially expressed genes associated with *M. nipponense* immune responses against DIV1 infection.

Category or Gene ID	Gene Description	Log2 FC
C-type lectin receptor signaling pathway		
TRINITY_DN2095_c0_g1	Ras	2.14
TRINITY_DN7682_c0_g2	lectin	10.98
TRINITY_DN758_c0_g1	C-type lectin-like	1.01
TRINITY_DN763_c0_g2	C-type lectin	2.72
TRINITY_DN39944_c0_g1	rho-related GTP-binding protein RhoA-C-like	10.72
TRINITY_DN3722_c0_g2	Protein kinase domain	1.60
TRINITY_DN8278_c0_g1	calmodulin-beta-like	−1.16
TRINITY_DN350_c0_g1	calmodulin	−12.15
Antigen processing and presentation		
TRINITY_DN3715_c0_g1	heat shock protein 70	2.28
TRINITY_DN3715_c2_g1	70 kD heat shock protein form 1	2.62
TRINITY_DN13116_c0_g1	heat shock protein	2.29
TRINITY_DN83_c0_g1	calnexin	5.76
TRINITY_DN17419_c0_g1	gamma-interferon-inducible lysosomal thiol reductase	1.78
TRINITY_DN37377_c0_g1	gamma-interferon-inducible lysosomal thiol reductase-like	3.18
TRINITY_DN19149_c0_g1	putative gamma-interferon-inducible lysosomal thiol reductase-like isoform X1	11.45
TRINITY_DN2412_c0_g2	gamma-interferon-inducible lysosomal thiol reductase-like	4.92
TRINITY_DN13933_c0_g1	cathepsin L	1.08
TRINITY_DN608_c0_g1	cathepsin B	−3.80
TRINITY_DN1606_c0_g1	cathepsin B	−3.37
Complement and coagulation cascades		
TRINITY_DN14365_c0_g1	trypsin	4.28
TRINITY_DN8140_c0_g1	trypsin-1-like isoform X1	3.26
TRINITY_DN3116_c0_g1	EGF-like domain	−1.83
TRINITY_DN39290_c0_g1	coagulation factor X	−11.19
Phagosome		
TRINITY_DN10442_c0_g1	V-type proton ATPase e subuni	1.08
TRINITY_DN163_c0_g1	V-type proton ATPase 21 kDa proteolipid subunit	1.03
TRINITY_DN83_c0_g1	calnexin	5.76
TRINITY_DN472_c0_g3	cytoplasmic-like	1.044
TRINITY_DN2887_c0_g1	integrin beta-PS-like	4.82
TRINITY_DN7473_c0_g1	integrin	11.76
Lysosome		
TRINITY_DN536_c0_g1	natural resistance-associated macrophage protein 2-like	10.28
TRINITY_DN563_c0_g1	formylglycine-generating enzyme-like	2.28
TRINITY_DN163_c0_g1	V-type proton ATPase 21 kDa proteolipid subunit	1.03
TRINITY_DN3997_c1_g1	glucosylceramidase-like	−1.70
TRINITY_DN547_c0_g1	alpha-N-acetylgalactosaminidase	−2.28
Apoptosis		
TRINITY_DN1075_c0_g2	caspase	4.72
TRINITY_DN9284_c0_g1	caspase	2.83
TRINITY_DN10429_c0_g1	caspase-3-like	3.10
TRINITY_DN2307_c0_g1	caspase 4	2.18
AMPK signaling pathway		
TRINITY_DN3645_c0_g1	calcium/calmodulin-dependent protein kinase type 1B-like	2.35
TRINITY_DN4459_c0_g1	serine/threonine-protein kinase mTOR-like	1.35
TRINITY_DN6500_c0_g1	serine/threonine-protein phosphatase 2A regulatory subunit B″ subunit delta-like isoform X2	1.19
TRINITY_DN8478_c0_g1	ras-related protein Rab-8B-like isoform X1	1.74
TRINITY_DN3645_c0_g1	calcium/calmodulin-dependent protein kinase type 1B-like	2.35
TRINITY_DN2871_c0_g1	elongation factor 2	1.24
TRINITY_DN14309_c0_g1	elongation factor 2	2.90
TRINITY_DN14309_c0_g2	elongation factor 2	2.93
TRINITY_DN1265_c0_g1	Rab10	1.33
Toll-like receptor signaling pathway		
TRINITY_DN168_c0_g1	Toll interacting protein	12.99
TRINITY_DN1659_c0_g1	ras-related C3 botulinum toxin substrate 1-like isoform X5	2.47
Wnt signaling pathway		
TRINITY_DN40081_c0_g1	Wnt6	10.39
TRINITY_DN31_c0_g3	casein kinase II subunit alpha	2.04
TRINITY_DN3956_c0_g1	serine/threonine-protein kinase NLK2-like	1.35
TRINITY_DN618_c0_g1	calcium/calmodulin-dependent protein kinase type II alpha chain-like	11.94
Toll and Imd signaling pathway		
TRINITY_DN6071_c0_g1	dorsal	8.64
TRINITY_DN1816_c0_g2	relish	2.51
TRINITY_DN4154_c0_g1	ubiquitin-conjugating enzyme E2-24 kDa	2.85

## Data Availability

The transcriptome data in this study are available in NCBI Sequence Read Archive (SRA) repository under BioProject accession number PRJNA1086475.
